# Factors and determinants of primary care to tertiary care referrals in Singapore: A multi-centre analysis using artificial intelligence-powered large language models

**DOI:** 10.1371/journal.pone.0338085

**Published:** 2026-02-05

**Authors:** Sky Wei Chee Koh, Jasper Yi Xuan Huang, Joelle Lam, Si Hui Low, Jun Cong Goh, Ge Ji, Xin Jin, Howard Bauchner, Yii Jen Lew

**Affiliations:** 1 National University Polyclinics, National University Health System, Singapore; 2 Yong Loo Lin School of Medicine, National University of Singapore, Singapore; 3 Faculty of Medicine, Nursing and Health Sciences, Monash University, Australia; 4 School of Physical and Mathematical Sciences, Nanyang Technological University, Singapore; 5 Data Science & Analytics, Alexandra Hospital, National University Health System, Singapore; 6 Boston University Chobanian & Avedisian School of Medicine, Boston, Massachusetts, United States of America; Universiti Malaysia Sabah, MALAYSIA

## Abstract

**Background:**

As Singapore adopts a population health approach under Healthier Singapore (Healthier SG), optimizing healthcare resources is crucial. We examined referral reasons (using large language models [LLM]), wait times, and analyse factors affecting referrals from primary to tertiary care.

**Methods:**

In 2023, 1,063,646 patient visits from seven primary care clinics in Singapore were analysed. Patient demographics, clinic, physician characteristics, referral volumes and wait times were extracted. LLM Claude 3.5 Sonnet was utilized to identify and classify top referral reasons within the most frequently referred specialties based on referral notes. Chi-square tests identified differences in referral rates among categorical variables, while a generalised linear model (GLM) with an identity link (normal distribution) determined factors influencing referrals by physicians.

**Findings:**

Around 1 in 5 visits resulted in a referral (n = 210,839, 19.8%), achieving 76.0% attendance rate. Referrals peaked among patients aged 60–70 years. Male (Odds ratio [OR] 0.88, 95% Confidence interval [CI] 0.87–0.89) and Malay (OR 0.71, 95% CI 0.70–0.72, compared with Chinese) patients were less likely to be referred. Significant variations were observed among clinics (p < 0.001). Ophthalmology (11.1%), orthopaedic surgery (10.3%), and emergency (10.0%) were the most referred specialties, with blurred vision (n = 7,461), abnormal diabetic retinopathy screening (n = 5,266) and pregnancy and antenatal care (n = 3,959) being the top referral reasons. 51.5% were routine referrals. Wait time averaged 52.7 days with 48.9% meeting targets, with long wait times for Gastroenterology & Hepatology, and Endocrinology. On average, each additional year of physician experience was associated with a reduction of 4.45 referrals per physician (95% CI: 1.40–7.58, p = 0.005).

**Interpretation:**

Our study highlighted disparities in referrals rates, patterns, and wait times. Continuing education and support for primary care is paramount. Resource allocation should be tailored to meet the population needs, with further research needed to ensure timely and appropriate referrals.

## Introduction

As gatekeepers of the healthcare system, primary care plays a pivotal role in optimising healthcare resource allocation for the population and regulating referrals to tertiary care [1]. In Singapore, primary care is delivered through a mix of public and private providers, including polyclinics and private general practitioner (GP) clinics. While polyclinics are government-run and provide subsidized care, private GPs operate independently, offering a range of services to patients. Referrals from primary care to tertiary care are a crucial aspect of the healthcare system, with patients typically requiring a referral from a primary care physician to access subsidized specialist care in hospitals. These referrals are available at all polyclinics and, for eligible patients, at participating private GPs under the Community Health Assist Scheme (CHAS).

However, the referral processes in several countries, including Singapore, is plagued by multiple issues that compromises on care delivery, quality, and timeliness [2,3]. One concern is the increasing demand for specialist care over the years, leading to a growing number of referrals from primary to tertiary care and lead to prolonged waiting times [4]. A study in Canada illustrated the extent of this issue, reporting a mean wait time of 60.1 days, with some specialties experiencing wait times of up to 168.5 days [5]. Furthermore, a notable proportion of referrals are inadvertently deemed inappropriate or unnecessary, as primary care physicians often navigate the challenge of balancing patient expectations with clinically indicated referrals, placing additional burden on already strained resources [6]. Disparities in referral patterns also exist, with certain populations facing unequal access due to socioeconomic status, ethnicity, and geographic location [7–9]. These issues can lead to severe consequences, including poorer outcomes, and increased costs, as well as patient dissatisfaction and no-shows [10–14].

Singapore’s referral system ensures patients receive subsidized specialist care by consulting primary care physicians first [15]. However, there has been an increasing trend of referrals from primary to tertiary care, increasing strain on hospital services. In response, the Singapore government introduced Healthier Singapore (Healthier SG) in July 2023, a multi-year healthcare reform to address problems of an ageing population, care fragmentation, growing disease burden, and rising healthcare costs [16]. Healthier SG aims to establish primary care as the foundation of Singapore’s healthcare, with patients enrolling with a primary care physician for lifelong, whole-person care, shifting from treatment to prevention and community-focused health management [17]. To assess its effectiveness, it is essential to understand the current state of referrals. Local studies showed referral inequities and quality concerns: ethnically malay patients were disproportionately referred to nephrology at lower estimated glomerular filtration rate (eGFRs), with higher metabolic disease burden and more missed appointments, while approximately 12% of emergency referrals were unnecessary, often for burns or smoke inhalation, with longer lengths of stay and higher likelihood when referred across healthcare clusters [18,19]. These studies were single-site, limited in scope, coming from angles of tertiary care institutions, and offered little visibility into referral volume, timeliness, appropriateness, and equity at a system level. Unlike countries with robust primary care systems [20,21], Singapore lacks comprehensive referral data, such as publicly available information on wait times and reasons for referral, suggesting the need for a multi-centre, large-scale analysis. Leveraging artificial intelligence (AI), specifically large language models (LLMs), to process high volumes of referral notes can standardize and efficiently classify referral reasons, enabling analyses that would be infeasible via manual review, which has not been previously conducted on a large scale basis. Key performance measures, such as whether referrals are seen within recommended timeframes, practice-level variation, and benchmarking of equity and appropriateness, are not available in current local datasets. While primary care physicians can assess referral urgency based on condition severity, limitations in data availability and patient scheduling dynamics hinder tracking of referral timeliness. This lack of data also hinders establishing a physician referral threshold, due to varying referral rates among primary care practices due to different practice and patient characteristics [22], and a lack of consensus on appropriate referrals [23].

To address these challenges, we aim to examine the patterns and characteristics of referrals from primary care clinics to tertiary care using AI to assist in our work, identify demographic and specialty-specific trends, analyse referral burden, including wait times and impact on healthcare services, and investigate the relationship between referral rates and wait times across different specialties. Additionally, we will attempt to determine factors affecting the number of referrals made by primary care physicians. In doing so, we can better understand the needs and burden of the population, ultimately informing strategies to optimize the referral system, reduce unnecessary referrals, and better utilise specialist appointments in the time of Healthier SG.

## Methods

### Setting

Singapore’s public primary healthcare system consists of 26 large, community-based polyclinics, offering subsidized primary care services for acute and chronic conditions, maternal and child health, and diagnostic, pharmacy, preventive, and allied health services. National University Polyclinics (NUP) is one of the three public primary healthcare institutions which operates seven polyclinics located across Western Singapore.

### Data sources

This cross-sectional, registry-based study utilized anonymized data from NUP’s medical records database (NUP Datamart). Experienced staff, independent of the study team, performed initial data extraction, validation, and quality control. During data cleaning and processing, missing values primarily in order case notes were identified, where physicians occasionally omitted prose descriptions of patients’ conditions and comorbidities, but had already selected referral reasons in multiple-choice fields. To address this, orders with selected multiple-choice fields were first reviewed and subsequently included in analysis, as these captured referral reasons within the multiple-choice fields. For orders with missing referral reasons in both multiple-choice fields and order notes, we applied listwise deletion, as these cases would likely be uninterpretable and were potentially rejected referrals, ensuring data quality before final analysis.

### Study population and variables

We included all doctor consultations for patients of all ages that resulted in outgoing referral orders to hospitals from 01/01/2023–31/12/2023, capturing comprehensive data on patient demographics (age, race, gender), consultation details (visit diagnoses, referral reason, referred specialty and hospital), visit and appointment dates, and referral order notes. We collected data on primary care physicians’ indicated referral priority, which was categorized into four groups: Immediate or Direct Admission, Fast Track and Direct Access (1–2 weeks), Early (<4 weeks), and Routine and Open Access (<8 weeks). Data on referring physician characteristics, including number of years of clinical experience, training location (overseas or local), main clinic of practice, and accreditation as family physicians (primary care physicians who have been board certified after completion of post-graduate specialty training) were also extracted. The following patients were excluded from the study: those with missing or incomplete data, duplicate referrals resulting from amendments due to changes in referral location, referrals rejected by hospitals, and referrals to non-hospital facilities, including community clinics and private specialist clinics. For wait time calculations, we measured the time interval between the date of referral by the primary care physician and the date of the patient’s specialist appointment. To ensure accuracy, we excluded patients who cancelled, had existing appointments, were uncontactable, deceased, had rejected referrals, or were no-shows from our wait time calculations, focusing on patients who successfully received and attended their scheduled appointments. We also excluded patients referred to the emergency departments, as these referrals typically occurred on the same day and bypassed the standard scheduling and appointment booking process.

As this study included all referrals made in 2023, sample size calculation was not required. Data was extracted on 25/07/2024 for research purposes.

### LLM classification

We first identified the top 10 most referred specialties. To analyse referral reasons from free-text referral notes, we used Bot-NUHS, an in-house large language model (LLM) hosted on Amazon Web Services and based on Claude 3.5 Sonnet, to read, interpret, and classify referral order notes. An integrated classification assistant (Classify-X) mapped each note to predefined categories aligned with the original referral labels. For example, Cardiology categories included chest pain, breathlessness, abnormal electrocardiogram (ECG) findings, palpitations, and heart murmurs. Single-label assignment was enforced so that each referral was classified into one reason only.

The LLM classification was run independently by two research team members (SHL and JCG) to ensure replicability, with discrepancies reviewed jointly and adjudicated by a senior primary care researcher (SWCK) to achieve consensus. For quality assurance, a random sample of 100 case notes were audited to verify correct referral reason classification. For unclear results, the LLM analysis was rerun. The top 20 referral reasons were then condensed into the top 10, with others grouped together given their smaller percentages. For each of the top 10 specialties, we repeated this process to identify their top 10 referral reasons; the top three were showcased in Table 2, with the remainder listed in S1 Table.

### Statistical analyses

Rstudio (R version 4.2.0), IBM SPSS Statistics Version 29.0, and Microsoft Excel 2010 were used in data cleaning and analysis. A *p*-value of <0.05 in the two-sided test was considered statistically significant. Descriptive statistics were performed, with numerical variables represented as mean with standard deviations (SD) for normally-distributed variables, median with inter-quartile range (IQR) for non-normal variables, and n (%) for categorical variables. Chi square tests and its corresponding odds ratios (ORs) were used to examine relationships between categorical variables (e.g., referral rate by patient race, gender, and clinic visited).

To identify factors associated with the number of referrals made by each physician, we fitted a generalized linear model (GLM) with an identity link and normal error distribution. Because physicians are nested within clinics, we initially specified a mixed-effects model with a random intercept for clinic to account for potential clustering, alongside fixed effects for years of clinical experience, number of patients seen, family physician accreditation, and training location. Residents were grouped separately from clinics due to their rotating clinic assignments during training. Model selection was guided by information criteria (Akaike Information Criterion [AIC] and Bayesian Information Criterion [BIC]), the magnitude and significance of the random-intercept variance, the intraclass correlation coefficient (ICC), and the conditional R-square. If clinic-level clustering proved negligible and fixed-effect estimates were materially unchanged, we adopted the simpler linear model as primary, using standard errors robust to clinic-level clustering. A normal specification because the referral counts of physicians were moderately large with minimal zero inflation, and model diagnostics (Q–Q plots, Shapiro–Wilk tests, and residual-vs-fitted plots) supported approximate normality of residuals and homoscedasticity; moreover, the identity link yielded estimates that were directly interpretable as absolute differences in referral counts. Exposure was accounted for by including number of patients seen as a co-variate in the normal-link model. We used simultaneous entry and fitted a full, pre-specified model with all covariables included in a single block. To correct for multiple testing, the Least Significant Difference (LSD) correction was applied. To ensure the stability of the model, we assessed multicollinearity among the independent variables using variance inflation factor (VIF) statistics, with a cutoff of VIF > 5 indicating significant collinearity. Results are presented as estimated coefficients (B), 95% confidence intervals (CIs), and corresponding p-values.

To ensure the robustness of our results and account for the count nature of the outcome, we conducted sensitivity analyses using Poisson and negative binomial GLMs with a log link to model referral rates per physician. However, the Poisson model demonstrated substantial over-dispersion with markedly higher AIC and BIC values, indicating poor fit. Therefore, only the results from the negative binomial GLM – which accounted for over-dispersion – were presented, and expressed as incidence rate ratios (IRRs), 95% CIs, and p-values (S1 Table).

### Ethics approval

The research was conducted in accordance with the Declaration of Helsinki national and institutional standards and approved by the National Healthcare Group (NHG) Domain-Specific Review Board (DSRB) in February 2024 (2024/00001). A waiver of patient consent was obtained, as this retrospective study utilized anonymized, registry-based data, posing minimal risk to participants, and having no potential impact on their clinical management, which had already been completed.

## Results

**Fig 1:**
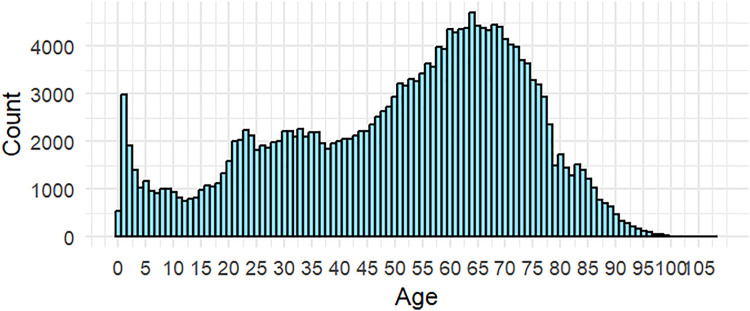
Referral Numbers by Age.

Our results revealed that nearly 1 in 5 patient visits (n = 210,839, 19.8%) resulted in a referral to tertiary care in 2023. Among unique patients, the average number of referrals per patient was 1.71 (SD 1.0) (Table 1). The referred patient population had a median age of 55.5 years (IQR 34.4,68.1) and an average of 1.71 referrals per patient (Table 1). Referrals increased markedly in the first year of life, followed by a gradual decline throughout early adolescence (Fig 1). A steady, upward trend then ensued, ending with a peak among patients aged 60–70 years. Subsequently, referrals decreased steadily among elderly patients. Majority of referred patients were Chinese (73.3%) and female (53.1%) (Table 1). Male patients (OR 0.88, 95% CI: 0.87–0.89) and those of Malay ethnicity (OR 0.71, 95% CI: 0.70–0.72) were significantly less likely to be referred. Furthermore, substantial variability in referral rates existed across clinics (p < 0.001), with Clinic B exhibiting the highest referral rate (23.1%), with significantly increased odds of referral (OR 1.14, 95% CI: 1.13–1.15) compared to other clinics. Of note, 83.9% of all referrals were made to hospitals within the same healthcare cluster.

**Table 1:** Descriptive statistics of referral characteristics.

Referral Characteristics	N = 210,839 (19.8%)	
Mean Age (SD)	50.9 (22.3)	
Median Age (IQR)	55.5 (34.4,68.1)	
Mean Number of Referrals per patient (SD)	1.71 (1.0)	
	**Number of Referrals (n, %)**	**Referral Rate (%)**
Race		
Chinese	154,619 (73.3)	20.6
Malay	24,124 (11.5)	15.5
Indian	18,732 (8.9)	20.4
Others	13,364 (6.3)	20.5
Gender		
Female	112,047 (53.1)	20.9
Male	98,792 (46.9)	18.8
Referring Clinic		
Clinic A	38,665 (18.3)	19.9
Clinic B	26,530 (12.6)	23.1
Clinic C	23,837 (11.3)	17.6
Clinic D	29,801 (14.1)	19.8
Clinic E	34,345 (16.3)	17.9
Clinic F	33,980 (16.1)	20.2
Clinic G	23,681 (11.3)	21.9
Referred Institution		
Within healthcare cluster	176,910 (83.9)	–
Cluster A	26,391 (12.5)	–
Cluster B	7,414 (3.5)	–
Others	124 (0.1)	–

Table 2 presents an overview of the top specialties referred, referral reasons, and referral priority. The top specialties referred to were ophthalmology (11.1%), orthopaedic surgery (10.3%), and emergency medicine (10.0%). Blurred vision (n = 7,461) and abnormal diabetic retinopathy screening (n = 5,266) were the top two reasons for referrals, collectively accounting for over half (53.9%) of all referrals to ophthalmology alone. Orthopedic surgery referral reasons were primarily categorized by anatomical location rather than specific diagnoses or conditions. The most common reasons for referrals to Emergency departments were cardiovascular concerns (n = 3,875, 18.5%), trauma and fractures (n = 2,840, 13.5%), and ophthalmological emergencies (n = 2,264, 10.8%). Notably, routine referrals accounted for 51.5% (n = 108,572) of all referrals (Table 2).

**Table 2:** Top 3 referral specialties, referral reasons, and referral priorities.

Top 3 Specialties Referred and Referral Reasons	n (%)	
**Ophthalmology**	**23,352 (11.1)**	
Blurred vision	7,461 (31.6)	
Abnormal diabetic retinopathy screening	5,266 (22.3)	
Floaters	1,988 (8.4)	
Cataract	1,607 (6.8)	
Glaucoma concerns	788 (3.3)	
Red eye	713 (3.0)	
Lid swelling	543 (2.3)	
Dry eyes	441 (1.9)	
Retinal issues	269 (1.1)	
Ptosis	225 (0.9)	
Others	4,292 (18.2)	
**Orthopedic Surgery**	**21,672 (10.3)**	
Foot and ankle conditions	3,158 (14.6)	
Back pain	2,968 (13.7)	
Osteoarthritis	2,877 (13.3)	
Spine issues	2,459 (11.4)	
Sports injuries	2,314 (10.7)	
Shoulder problems	2,090 (9.6)	
Knee pain	1,535 (7.1)	
Neurological symptoms	1,112 (5.1)	
Fractures	915 (4.2)	
Soft tissue abnormalities	576 (2.7)	
Others	1,668 (7.7)	
**Emergency Medicine**	**20,998 (10.0)**	
Cardiovascular concerns	3,875 (18.5)	
Trauma and fractures	2,840 (13.5)	
Ophthalmological emergencies	2,264 (10.8)	
Infectious diseases	2,219 (10.6)	
Neurological symptoms	1,946 (9.3)	
Abdominal pain and gastrointestinal problems	1,777 (8.5)	
Respiratory issues	1,349 (6.4)	
Dermatological conditions	822 (3.9)	
Urological and renal issues	726 (3.5)	
Gynaecological and obstetric emergencies	527 (2.5)	
Others	2,653 (12.6)	
	
**Referral Priority**	**n (%)**	**% referrals within target**
Immediate or Direct Admission	22,506 (10.7)	–
Fast Track and Direct Access (1–2 weeks)	29,890 (14.2)	44.2
Early (<4 weeks)	49,871 (23.6)	43.4
Routine and Open Access (<8 weeks)	108,572 (51.5)	52.9

**Fig 2:**
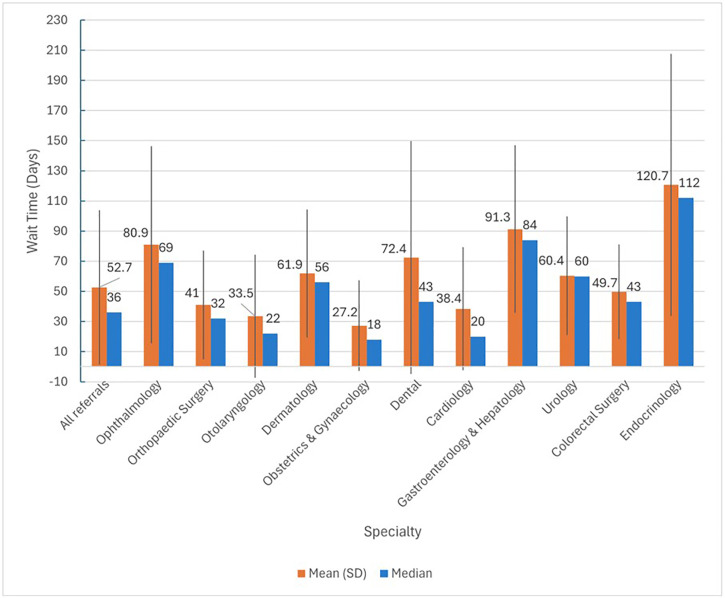
Mean and Median Referral Wait Times by Specialty.

**Fig 3:**
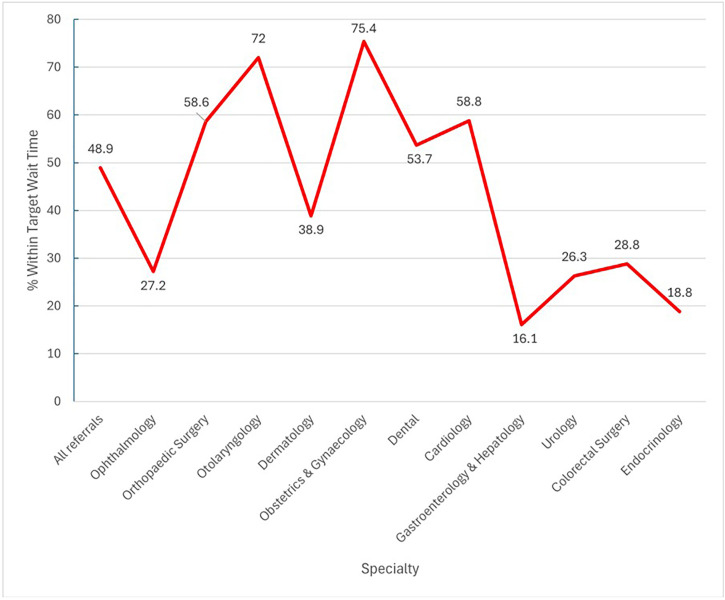
Percentage of Referrals within Target Wait Time by Specialty.

76.0% (n = 160,140) of scheduled referrals were attended by patients. The percentage of referrals meeting their expected wait time was higher for routine referrals (52.9%) (Table 2). The average wait time to appointment after referral was 52.7 (51.2) days, with 48.9% meeting targets (Table 3). Among the top 10 most referred specialties, Gastroenterology & Hepatology had the longest mean (91.3) and median (84.0) referral wait times, with only 16.1% meeting stipulated targets. Other specialties experiencing prolonged wait times included ophthalmology, dental, dermatology, and urology. Interestingly, dental appointments had a mean wait time of 72.4 days, but a median wait time of 43.0 days, indicating a skewed distribution with some patients facing extremely long wait times (Fig 2 and 3). Among specialties outside the top 10 most referred, endocrinology (n = 2,299) had the longest referral wait times. The mean wait time was 120.7 days (SD: 86.9), with a median wait time of 112.0 days (IQR: 49.0–151.5). Only 18.8% of referrals to endocrinology met the requested appointment times (Fig 2 and 3).

**Table 3:** Referral wait times for specialties.

Specialties	Referral wait Times		% Referrals within target wait time
	Mean (SD)	Median (IQR)	
All referrals	52.7 (51.2)	36.0 (16.0–73.0)	48.9
Ophthalmology	80.9 (65.3)	69.0 (26.0–125.0)	27.2
Orthopedic Surgery	41.0 (36.1)	32.0 (15.0–55.0)	58.6
Otolaryngology	33.5 (40.9)	22.0 (11.0–38.0)	72.0
Dermatology	61.9 (42.4)	56.0 (32.0–87.0)	38.9
Obstetrics & Gynecology	27.2 (30.1)	18.0 (10.0–31.0)	75.4
Dental	72.4 (77.3)	43.0 (17.0–98.0)	53.7
Cardiology	38.4 (40.9)	20.0 (9.0–56.0)	58.8
Gastroenterology & Hepatology	91.3 (55.6)	84.0 (50.0–128.0)	16.1
Urology	60.4 (39.4)	60.0 (29.0–86.0)	26.3
Colorectal Surgery	49.7 (31.5)	43.0 (28.0–65.0)	28.8
Endocrinology	120.7 (86.9)	112.0 (49.0–151.5)	18.8

The results of our GLM are presented in Table 4. Comparing the mixed-effects model (clinic random intercept) with the standard linear model indicated negligible clinic-level clustering and superior fit for the simpler specification. For the mixed-effects model, AIC was 4443.37 and BIC was 4450.89; marginal R-square was 0.82 (fixed effects) and conditional R-square was 0.839 (fixed and random effects), with a low ICC (0.019), and the clinic random-effect variance was small and non-significant (variance 5340.22, SE 3675.97, p = 0.146). Residual variance was 45726.911 (SE 3638.419, p < 0.001). In contrast, the standard linear model achieved lower information criteria (AIC 4357.53; BIC 4361.28), a slightly higher pseudo-R square (0.848), with a similar residual variance (45718.276; SE 3637.151; p < 0.001). Fixed-effect estimates were materially unchanged across specifications. Given the minimal ICC and non-significant random intercept coupled with better parsimony and fit, we presented the standard linear model in our final analysis. VIF values for variables ranged between 1.04 to 1.63, below the cut off threshold of VIF > 5. The GLM showed significant associations between the number of referrals and the number of patients seen (p < 0.001), and number of years of clinical experience (p = 0.005). Conversely, training location and accredited family physician status showed no significant associations with referral numbers. Every additional patient seen was associated with an increase of 0.184 referrals (95% CI: 0.174–0.194). After adjusting for clinic, family physician accreditation, training location, and patients seen, every additional year of clinical experience was associated with a reduction of 4.45 referrals per physician (95% CI: 1.40–7.58, p = 0.005).

**Table 4:** Mixed-effects generalized linear model of factors affecting referrals to tertiary care.

Referrals	*p*	β	95% CI	
			Lower	Upper
Number of Years of Clinical Experience	**0.005**	−4.448	−7.580	−1.397
Number of Patients Seen	**<0.001**	0.184	0.174	0.194
Physicians who trained locally (ref = overseas)	0.65	−11.2	−60.0	37.6
Non-family physicians (ref = family physicians)	0.52	−20.9	−85.3	43.5
Residents without fixed clinic*	**0.036**	−113.5	−219.7	−7.38
Clinic A*	0.39	−45.1	−148.2	57.9
Clinic B*	0.33	62.3	−63.6	188.3
Clinic C*	0.25	−66.1	−178.5	46.2
Clinic D*	0.48	−40.2	−152.0	71.6
Clinic E*	**<0.001**	−215.2	−327.4	−103.0
Clinic F*	0.92	−5.48	−119.0	108.0

*Ref = Clinic G.

Dependent variable: number of referrals to tertiary care per physician during the study period. Coefficients (β) represent the absolute change in the expected number of referrals associated with a one-unit increase in the predictor (or the difference versus the reference category for categorical variables), holding other variables constant and accounting for clinic clustering.

Our sensitivity analyses fitted a Poisson and negative binomial GLM with a log link and offset for patient volume. The Poisson model showed poor fit (AIC 37138.85, BIC 37183.38), marked overdispersion on diagnostic checks, and did not improve inference relative to the primary linear model. The negative binomial GLM demonstrated improved model fit close to the linear model (AIC 4483.86, BIC 4532.01), with improved dispersion properties, and showcased in S2 Table. While number of patients seen was positively associated with referral rate, with each additional 100 patients seen associated with a 4.2% higher referral rate (IRR 1.042, 95% CI 1.038–1.045, p < 0.001), number of years of clinical experience did not significantly affect referral counts.

## Discussion

Our study’s findings highlighted a significant burden of referrals from primary to tertiary care, with substantial variability in referral rates across diverse patient demographics, clinics, and specialties. It also revealed distinct patterns, including referral reasons, priority, and wait times. The GLM findings indicated that clinical experience significantly predict referral numbers among primary care physicians, after adjusting for number of patients seen. This was not significant in our negative binomial sensitivity analyses, and we attributed it to the fact that years of experience appeared as an additive (roughly constant change across the range) rather than multiplicative, where the negative binomial structure shrinks toward IRRs close to 1 and reduces power. The inverse relationship between physician experience and referral numbers were also observed in several studies, which may be attributed to more experienced physicians’ ability to prioritize issues, engage in shared decision-making, and manage higher-risk and complex cases within primary care [24–26]. This expertise, built through increased continuity of care and patient understanding, reduces the need for referrals [27,28]. However, this relationship may be influenced by patient volume, which our study found to significantly increase referral numbers. This is consistent with other research, where high patient volumes led to higher referral volumes among GPs, potentially due to burnout, reduced decision-making capacity, and cognitive overload [29,30]. To ensure good quality of patient care and health system, healthcare organizations may need to consider strategies to manage patient volume and support physician training and collaboration. Future research should evaluate the teamlet care model in Singapore polyclinics to determine whether a predominantly two-physician, one care manager and one care coordinator structure improves care continuity and, in turn, lowers referral rates [31].

Our analysis revealed that male and Malay patients were less likely to be referred to tertiary care, suggesting differences in health beliefs and health-seeking behavior [32]. This disparity is consistent with multiple studies conducted in Southeast Asia which showed that ethnic Malays had reduced participation rates in medical check-ups and cancer screenings, with more emotional and financial barriers to accessing further care [33–36]. This association should be interpreted cautiously because our study did not adjust for key potential confounders such as socioeconomic status (e.g., income, education, housing type), comorbidity burden, insurance or subsidy coverages, language proficiency, and health literacy, which influences referral to specialty care [37–40]. In contrast, females were found to have increased attendances and referrals. This pattern aligns with the literature showing that women generally exhibited higher healthcare utilization [41,42], possibly due to reduced thresholds for seeking care, preventive and reproductive care needs [40,43], while higher referrals to specialist care may be due to perceived unmet care needs [44]. Future work could stratify referral patterns by comorbidity, healthcare utilization intensity, and other abovementioned factors to determine whether gender or ethnic differences persist after accounting for these influences. As Singapore implements its Healthier SG initiative, it is essential to address the needs of these underserved populations by promoting awareness, positive health-seeking behaviours, and health-promoting beliefs to ensure equitable access to healthcare. Furthermore, it is crucial to recognize that racial, ethnic, and gender disparities in healthcare access and utilization can evolve over time, influenced by healthcare reforms, and to adapt strategies accordingly [45].

Clinic B exhibited significantly higher referral rates compared to other clinics. One contributor was its staffing mix: our chi-square analysis found that the odds of Clinic B not being staffed by accredited family physicians were 3.37 times higher than in other clinics (OR 3.37, 95% CI 1.35–8.40, p = 0.014), excluding residents. While novelty (being the newest clinic) and several other factors, such as clinic characteristics, local patient demographics, and service availability could plausibly contribute, we did not have these data to evaluate these explanations. Further research is needed to explore the underlying reasons for this disparity.

Our referral rates were significantly higher compared to international benchmarks (9.3% in United States, 9.5% in United Kingdom) despite being countries with developed primary care systems [4,46]. Consistent with global trends [9,21], chest pain was the leading reason for referral in our study. While 76.0% of scheduled referrals were attended as scheduled, this was considered better than primary care systems in the United States, where 61.1% had documented appointment scheduling dates [47]. The remaining 24.0% were attributed to various reasons, including being uncontactable, cancellations, mortality while waiting, rejected referrals due to incorrect disposition, and no-shows. However, our analysis was limited by the inability to track patients who may have independently scheduled another appointment after an initial no-show; actual attendance rate is expected to be higher. Nevertheless, this highlights the need for further research and operational improvements to optimize resource utilization, reduce administrative inefficiencies, and enhance the overall referral process.

The top three non-emergency specialties receiving referrals were ophthalmology, orthopaedics surgery, and otolaryngology. Despite high volumes, orthopaedics and otolaryngology departments kept wait times relatively low, demonstrating efficient resource allocation. In contrast, ophthalmology, dermatology and dental experienced long wait times, with striking mean times in our sample (gastroenterology & hepatology: 91.3 days, and endocrinology: 120.7 days). Such delays can have important implications: reduced patient satisfaction, increased anxiety and no-show rates [47–49], and even potential harm through delayed diagnosis and treatment initiation [50,51]. Our study’s findings align well with a Canadian study, with comparable mean overall wait time of 60.1 days [5]. However, individual specialty wait times differed, likely due to availability of structured care paths, bypassing need for referrals, reducing wait time, and being excluded from referral counts. Singapore’s open access programmes, such as open access esophagogastroduodenoscopy, have successfully reduced wait times, with a median wait time of 23 days, compared to 84 days for a gastroenterologist appointment [52]. This programme allowed patients to have access to investigations and procedures without a prior specialist visit. Despite this, wait times for gastroenterology & hepatology remained high, indicating a need for further improvements and expansion of such open access services. Dental referrals exhibited a striking disparity between mean and median wait times, with the mean far exceeding the median and a standard deviation value higher than the mean wait times. This suggested that certain sub-specialty services within dentistry were experiencing exceptionally long wait times; strategies such as enhancing training for generalists to manage complex cases or re-evaluating the level of sub-specialization required for initial referrals can be considered to streamline access to care. While we sought to contextualize these waits against system expectations, comprehensive national wait-time targets stratified by specialty and urgency were not uniformly available; in practice, institutions apply triage categories that determine expected timelines. To mitigate risks when referral timing was incongruent with clinical urgency, establishing feedback loops to primary care through virtual professional consultations, systemic value-stream mapping to identify bottlenecks, or even providing adequate safety netting advice and explanation to patients could help ensure timely care [53–55].

The Healthier SG initiative is crucial in empowering primary care physicians to deliver effective patient care and reduce unnecessary referrals. By addressing factors influencing referral efficiency, such as individual patient factors, clinical decision making, technology, improved organizational management and quality monitoring, Healthier SG provides a good platform to optimise the referral process [56,57]. The existing referrals prioritization framework, which categorizes patients based on condition severity, ensures timely access to care for those in greatest need. This evidence-based approach, also adopted in Norway and Canada [58,59], relies on primary care physicians’ clinical acumen, promoting healthcare equity in referral patterns. The initiative’s emphasis on longitudinal care enables primary care physicians to develop a deeper understanding of their patients’ needs, fostering more informed decision-making and personalized care. Empowering and educating primary care physicians has yielded positive results, such as reducing unnecessary orthopedic referrals [60]. Simple interventions and quality improvement initiatives can significantly reduce referrals without incurring significant costs. By continuing to empower primary care physicians through the Healthier SG initiative, we can reduce referrals further. Ultimately, improving primary care physicians’ resources, knowledge, and training is key to reducing referrals, enhancing their decision-making abilities, and optimizing healthcare resource utilization.

As the first study on referrals in Singapore primary care, this study provides key insights into referral patterns. The comprehensive dataset, large sample size, and diverse patient demographics enabled the examination of trends, wait times, referral reasons, and disparities. Minimal missing data and robust statistical analysis using a GLM with good model fit added to the reliability of the findings. Sensitivity analyses using Poisson distribution further supported the results. Overall, the study’s findings are representative of the referral landscape across public primary care in Singapore, and have important implications for healthcare policy and practice, particularly in Singapore’s Healthier SG initiative.

This study has several limitations. Referral reasons extracted from free-text referrals may be inaccurate or inconsistent with International Classification of Diseases (ICD)-10 codes. We did not assess referral appropriateness, care complexity, or patient-initiated referrals, as these were outside the study’s scope and not available in our data. Future work incorporating standardized appropriateness criteria and richer clinical context would enable a more holistic evaluation. A key limitation is the exclusion of referrals from private CHAS general practitioners. CHAS encompasses a large segment of primary care in Singapore (at least 1,650 private GP clinics, with approximately 1.3 million eligible Singaporeans) [61], so omitting these referrals means our findings primarily reflect public-sector patterns and are not generalisable to the private GP sector, where access pathways (including direct-to-test options and private specialist appointments) can differ. Private GP referrals could materially shape specialty demand and wait-time distributions in Singapore, but these effects are not captured by our dataset and therefore cannot be attributed to our findings. We similarly excluded inter-specialty and emergency department referrals, which could lead to underestimation of overall referral volume and changes in the observed specialty mix. Listwise deletion of orders with missing referral notes may introduce selection bias. In our setting, such referrals are typically rejected and require physicians to recreate the order, so excluded records likely represent non-completed referrals, though residual bias remains. Of note, referrals to specialist outpatient clinics are primarily channelled through primary care physicians, with other subsidized referral pathways including emergency department visits and inter-specialty referrals within hospitals. Allied health professionals and nurses in primary care also refer patients through the same workflow, which may involve physician oversight, and are therefore included in this analysis. As an observational study, associations do not imply causation. Furthermore, the study could not determine the proportion of patient-initiated referrals, particularly for specialties with high referral numbers and prolonged wait times. The study also could not determine the underlying reasons for the observed associations between demographics and referral rates, warranting further qualitative research. Referral wait times are dynamic and may affect data accuracy. While exclusions (such as rejected referrals, amended duplicates, non-hospital referrals) improved data quality and analytic clarity, they may introduce selection bias if excluded cases differ systematically from included patients, potentially underestimate actual wait times, and omit patients affected by system inefficiencies. Lastly, our GLM, which was conducted at the individual physician level, did not account for crucial patient-related factors, such as age. This limitation highlights the need for additional research to further explore the complex interplay between patient characteristics, physician factors, and referral patterns.

## Conclusion

In conclusion, our study highlighted the importance of addressing disparities in referral patterns among clinics and specific patient groups in Singapore’s primary care setting. The inverse relationship between clinical experience and referral rates suggests that more experienced physicians are better equipped to manage patients within the primary care setting, underscoring the importance of investing in continuing medical education, support, and resources for primary care physicians. The variability in referral rates and wait times across different specialties suggests that there are opportunities for improvement in the allocation of resources and the delivery of specialist care. Further research is needed to ensure that referrals are timely, appropriate, and equitable, and that patients receive high-quality care that meets their needs.

## Supporting information

S1 Table Top 10 Referral Specialties and Reasons. (DOCX)

S2 Table Negative Binomial GLM of Factors Affecting Referral Count Per Physician to Tertiary Care. (DOCX)

S1 Data Primary to tertiary care referral records. (XLSX)

S2 Data Physician characteristics and referral rates. (CSV)
