# Supplementary material for: Factors and determinants of primary care to tertiary care referrals in Singapore: A multi-centre analysis using artificial intelligence-powered large language models
Source: PLoS One. 2026 Feb 5;21(2):e0338085. doi: 10.1371/journal.pone.0338085 (PMC12875445; doi:10.1371/journal.pone.0338085)
Supplement: S1 Table — (DOCX) [file pone.0338085.s001.docx]

**Table S1. Top 10 Referral Specialties and Reasons**

| **Top 10 Specialties Referred and Referral Reasons** | **n (%)** |
| --- | --- |
| **Ophthalmology** | **23,352 (11.1)** |
| Blurred vision | 7,461 (31.6) |
| Abnormal diabetic retinopathy screening | 5,266 (22.3) |
| Floaters | 1,988 (8.4) |
| Cataract | 1,607 (6.8) |
| Glaucoma concerns | 788 (3.3) |
| Red eye | 713 (3.0) |
| Lid swelling | 543 (2.3) |
| Dry eyes | 441 (1.9) |
| Retinal issues | 269 (1.1) |
| Ptosis | 225 (0.9) |
| Others | 4,292 (18.2) |
| **Orthopedic Surgery** | **21,672 (10.3)** |
| Foot and ankle conditions | 3,158 (14.6) |
| Back pain | 2,968 (13.7) |
| Osteoarthritis | 2,877 (13.3) |
| Spine issues | 2,459 (11.4) |
| Sports injuries | 2,314 (10.7) |
| Shoulder problems | 2,090 (9.6) |
| Knee pain | 1,535 (7.1) |
| Neurological symptoms | 1,112 (5.1) |
| Fractures | 915 (4.2) |
| Soft tissue abnormalities | 576 (2.7) |
| Others | 1,668 (7.7) |
| **Emergency Medicine** | **20,998 (10.0)** |
| Cardiovascular concerns | 3,875 (18.5) |
| Trauma and fractures | 2,840 (13.5) |
| Ophthalmological emergencies | 2,264 (10.8) |
| Infectious diseases | 2,219 (10.6) |
| Neurological symptoms | 1,946 (9.3) |
| Abdominal pain and gastrointestinal problems | 1,777 (8.5) |
| Respiratory issues | 1,349 (6.4) |
| Dermatological conditions | 822 (3.9) |
| Urological and renal issues | 726 (3.5) |
| Gynaecological and obstetric emergencies | 527 (2.5) |
| Others | 2,653 (12.6) |
| **Otolaryngology** | **15,916 (7.5)** |
| Hearing loss | 3,051 (19.2) |
| Nasal congestion | 1,562 (9.8) |
| Sleep disorders/snoring | 1,455 (9.1) |
| Tinnitus | 1,297 (8.1) |
| Ear pain/discomfort | 1,142 (7.2) |
| Vertigo | 1,010 (6.3) |
| Neck lumps/nodes | 985 (6.2) |
| Ear discharge | 980 (6.2) |
| Sore throat | 958 (6.0) |
| Epistaxis | 660 (4.2) |
| Others | 2,860 (18.0) |
| **Dermatology** | **14,537 (6.9)** |
| Skin lesions | 2,789 (19.2) |
| Eczema | 2,162 (14.9) |
| Rashes | 1,721 (11.8) |
| Warts | 1,220 (8.4) |
| Acne | 859 (5.9) |
| Hair loss | 832 (5.7) |
| Pigmentation issues | 720 (5.0) |
| Dermatitis | 697 (4.8) |
| Urticaria | 663 (4.6) |
| Fungal infections | 471 (3.2) |
| Others | 2,403 (16.6) |
| **Obstetrics & Gynecology** | **12,493 (5.9)** |
| Pregnancy and antenatal care | 3,959 (31.7) |
| Abnormal uterine bleeding | 1,666 (13.3) |
| Menstrual disorders | 1,335 (10.7) |
| Fertility issues | 780 (6.2) |
| Contraception management | 621 (5.0) |
| Cervical abnormalities | 619 (5.0) |
| Postmenopausal bleeding | 462 (3.7) |
| Ovarian cysts/masses | 416 (3.3) |
| Vulvar/vaginal disorders | 370 (3.0) |
| Fibroids | 348 (2.8) |
| Others | 1,832 (14.7) |
| **Dental** | **12,160 (5.8)** |
| Oral surgery | 2,651 (21.8) |
| Endodontic procedures | 2,085 (17.2) |
| Removable prosthetics | 1,129 (9.3) |
| Fixed prosthetics | 776 (6.4) |
| Periodontal care | 759 (6.2) |
| Prosthodontic treatments | 675 (5.6) |
| Orthodontics | 653 (5.4) |
| Urgent dental care | 650 (5.4) |
| Pediatric dentistry | 482 (4.0) |
| Temporomandibular disorders | 397 (3.3) |
| Others | 1,903 (15.7) |
| **Cardiology** | **9,249 (4.4)** |
| Chest pain | 1,826 (19.8) |
| Abnormal ECG/CXR findings | 1,801 (19.5) |
| Breathlessness | 1,128 (12.2) |
| Palpitations | 1,055 (11.4) |
| Atypical cardiac symptoms | 497 (5.4) |
| Heart murmur evaluation | 331 (3.6) |
| Arrhythmia management | 287 (3.1) |
| Screening for cardiovascular disease | 183 (2.0) |
| Syncope investigation | 149 (1.6) |
| Cardiovascular risk factor assessment | 148 (1.6) |
| Others | 1,841 (19.9) |
| **Gastroenterology & Hepatology** | **8,692 (4.1)** |
| Dyspepsia/GERD | 3,180 (36.6) |
| Abnormal liver function tests | 744 (8.6) |
| Non-alcoholic fatty liver disease | 677 (7.8) |
| Anemia | 635 (7.3) |
| Change in bowel habits | 609 (7.0) |
| Endoscopy procedures | 160 (1.8) |
| Dysphagia | 124 (1.4) |
| Family history of gastric cancer | 33 (0.4) |
| Weight loss | 33 (0.4) |
| Jaundice | 32 (0.4) |
| Others | 2,465 (28.4) |
| **Urology** | **8,032 (3.8)** |
| Male urological issues | 1,965 (24.5) |
| Microscopic hematuria | 1,289 (16.1) |
| Benign prostatic hyperplasia (BPH) | 912 (11.4) |
| Urinary tract infection (UTI) | 667 (8.3) |
| Gross hematuria | 442 (5.5) |
| Renal/ureteric colic | 429 (5.3) |
| Elevated prostate-specific antigen (PSA) | 354 (4.4) |
| Recurrent UTIs | 267 (3.3) |
| Kidney and ureteral stones | 68 (0.9) |
| Lower urinary tract symptoms (LUTS) | 62 (0.8) |
| Others | 1,577 (19.6) |
