# Supplementary material for: Factors and determinants of primary care to tertiary care referrals in Singapore: A multi-centre analysis using artificial intelligence-powered large language models
Source: PLoS One. 2026 Feb 5;21(2):e0338085. doi: 10.1371/journal.pone.0338085 (PMC12875445; doi:10.1371/journal.pone.0338085)
Supplement: S2 Table — (DOCX) [file pone.0338085.s002.docx]

**Table S2. Negative Binomial GLM of Factors Affecting Referral Count Per Physician to Tertiary Care**

| **Referrals** | β | *p* | IRR | 95% CI | |
| --- | --- | --- | --- | --- | --- |
|  |  |  |  | Lower | Upper |
| Number of Years of Clinical Experience | -0.007 | 0.13 | 0.993 | 0.983 | 1.002 |
| Number of Patient Seen (Per 100)^#^ | 0.041 | **<0.001** | 1.042 | 1.038 | 1.045 |
| Physicians who trained locally (ref = overseas) | 0.051 | 0.475 | 1.052 | 0.915 | 1.211 |
| Non-family physicians (ref = family physicians) | -0.173 | 0.060 | 0.841 | 0.702 | 1.007 |
| Residents without fixed clinic* | -0.263 | 0.084 | 0.769 | 0.570 | 1.037 |
| Clinic A* | -0.057 | 0.703 | 0.944 | 0.703 | 1.268 |
| Clinic B* | 0.206 | 0.263 | 1.229 | 0.856 | 1.764 |
| Clinic C* | -0.161 | 0.327 | 0.852 | 0.617 | 1.175 |
| Clinic D* | -0.148 | 0.363 | 0.862 | 0.627 | 1.187 |
| Clinic E* | -0.305 | 0.063 | 0.737 | 0.535 | 1.017 |
| Clinic F* | -0.129 | 0.434 | 0.879 | 0.637 | 1.214 |

*Ref = Clinic G
^#^ Variable scaled to per 100 patients

Dependent variable: referral count per physician to tertiary care . Coefficients (β) represent the change in the expected log referrals count with a one-unit increase in the predictor (or the difference versus the reference category for categorical variables), holding other variables constant. IRR (Incidence Rate Ratio) > 1 represents a higher expected referral rate.
